# Regulation of transcription termination by glucosylated hydroxymethyluracil, base J, in *Leishmania major* and *Trypanosoma brucei*

**DOI:** 10.1093/nar/gku714

**Published:** 2014-08-07

**Authors:** David Reynolds, Laura Cliffe, Konrad U. Förstner, Chung-Chau Hon, T. Nicolai Siegel, Robert Sabatini

**Affiliations:** 1 Department of Biochemistry and Molecular Biology, University of Georgia, Davison Life Sciences Building, 120 Green Street, Athens, GA 30602-7229, USA; 2 Core Unit Systems Medicine, University of Wuerzburg, Wuerzburg 97080, Germany; 3 Institut Pasteur, Unité Biologie Cellulaire du Parasitisme, Département Biologie cellulaire et infection, Paris 75015, France; 4 INSERM U786, Paris 75015, France; 5 Research Center for Infectious Diseases, University of Wuerzburg, Wuerzburg 97080, Germany

## Abstract

Base J, β-d-glucosyl-hydroxymethyluracil, is an epigenetic modification of thymine in the nuclear DNA of flagellated protozoa of the order Kinetoplastida. J is enriched at sites involved in RNA polymerase (RNAP) II initiation and termination. Reduction of J in *Leishmania tarentolae* via growth in BrdU resulted in cell death and indicated a role of J in the regulation of RNAP II termination. To further explore J function in RNAP II termination among kinetoplastids and avoid indirect effects associated with BrdU toxicity and genetic deletions, we inhibited J synthesis in *Leishmania major* and *Trypanosoma brucei* using DMOG. Reduction of J in *L. major* resulted in genome-wide defects in transcription termination at the end of polycistronic gene clusters and the generation of antisense RNAs, without cell death. In contrast, loss of J in *T. brucei* did not lead to genome-wide termination defects; however, the loss of J at specific sites within polycistronic gene clusters led to altered transcription termination and increased expression of downstream genes. Thus, J regulation of RNAP II transcription termination genome-wide is restricted to *Leishmania* spp., while in *T. brucei* it regulates termination and gene expression at specific sites within polycistronic gene clusters.

## INTRODUCTION

Members of the Kinetoplastida order include the human parasites *Trypanosoma brucei*, *Trypanosoma cruzi* and *Leishmania major*, which cause African sleeping sickness, Chagas’ disease and leishmaniasis, respectively. Kinetoplastids are early diverged protozoa with unique genome arrangements where genes are organized into long clusters, or polycistronic transcription units (PTUs), which are transcribed by RNA polymerase (RNAP) II (1–3). RNAP II transcription initiation and termination occurs at regions flanking PTUs called divergent strand switch regions (dSSRs) and convergent strand switch regions (cSSRs), respectively (4). Transcription also terminates and initiates at head-tail (HT) sites, where transcription of an upstream PTU terminates and transcription of a downstream PTU on the same strand initiates (5–7). Pre-messenger RNAs (mRNA) are processed to mature mRNA with the addition of a 5′ spliced leader sequence through *trans*-splicing, followed by 3′ polyadenylation (8–13). In other eukaryotes, RNAP II termination is generally coupled to 3′ mRNA processing (14). In kinetoplastids, however, 3′ mRNA processing is instead coupled to *trans*-splicing of the 5′ end of the adjacent gene (15), preventing premature termination within a PTU. The mechanism of RNAP II termination in kinetoplastids remains unknown.

While little is known regarding the regulation of gene expression in kinetoplastids, the unique genome arrangement and polycistronic transcription of functionally unrelated genes has led to the assumption that transcription is an unregulated process in these organisms and that most gene regulation occurs post-transcriptionally (16,17). Recently, however, numerous epigenetic markers have been found enriched specifically at sites of transcription initiation and termination, including histone methylation and acetylation, histone variants and base J, which could function to regulate gene expression at the level of transcription (5,6,18,19).

Base J, β-d-glucosyl-hydroxymethyluracil, is a thymidine modification found in kinetoplastids and closely related unicellular flagellates (20,21). J is largely a telomeric modification, but is also found internally within chromosomes at RNAP II transcription initiation and termination sites (18,22–25). Using high-throughput sequencing and chromatin immunoprecipitation (ChIP) analysis, we identified base J localized within dSSRs and cSSRs in the genomes of *T. brucei*, *T. cruzi* and *L. major* (18,26). Recent high-throughput sequencing studies in *L. major* and *L. tarentolae* confirmed this internal J localization at RNAP II transcription regulatory sites (27). Because base J is a conserved DNA modification specific to kinetoplastids (not present in the mammalian host) with a possible role in key regulatory processes, it represents a potential drug target to treat the diseases caused by these pathogens (28).

As reviewed in (28), base J is synthesized in a two-step pathway in which a thymidine hydroxylase, JBP1 or JBP2, hydroxylates T residues at specific positions in DNA to form hydroxymethyluracil (HOMedU), followed by the transfer of glucose to HOMedU by a glucosyltransferase (28). Both JBP1 and JBP2 belong to the new TET/JBP subfamily of dioxygenases, which require Fe^2+^ and 2-oxoglutarate (2-OG) for activity (29–31). The synthesis of base J can be inhibited by knocking out JBP1 and JBP2 or by competitive inhibition of the thymidine hydroxylase domain of JBP1 and JBP2 by dimethyloxalylglycine (DMOG), a structural analog of 2-OG (29,32,33). Removal of both JBP1 and JBP2 in *T. brucei* or growth in the presence of DMOG results in cells devoid of base J (J null) (29,34); however, studies thus far have not identified defects associated with the loss of J in *T. brucei*. In contrast, the inability to delete both JBP enzymes from *T. cruzi* or *Leishmania* spp. suggests the modification is essential in these organisms (26,35).

Base J reduction in *T. cruzi*, following the deletion of either JBP1 or JBP2, resulted in the formation of more active chromatin at transcription initiation sites, increased RNAP II recruitment and PTU transcription rate, and global changes in gene expression (26,36). The loss of J at cSSRs had no detectable effect on RNAP II transcription termination at the end of PTUs (26). The specific J-dependent loss of nucleosomes at dSSRs and not cSSRs is consistent with a unique role of J in regulating RNAP II initiation in *T. cruzi* (36). However, van Luenen *et al.* recently found that reduction of base J in a JBP2 KO cell line of *Leishmania tarentolae*, a pathogen of lizards, resulted in the generation of antisense RNAs (27). Treating the JBP2 KO cells with 0.6-μM bromodeoxyuridine (BrdU) further reduced J by an unknown mechanism and resulted in increased levels of antisense small RNAs and cell death. It was argued that J loss resulted in readthrough transcription at cSSR termination sites, which led to the production of RNAs antisense to the genes on the opposite strand. Direct evidence of readthrough transcription was not shown however, and it remains possible that antisense RNAs were produced through RNAP reinitiation within the cSSR, as opposed to readthrough transcription. Additionally, despite the known toxicity of BrdU to eukaryotic cells (37–39), it was suggested that massive transcriptional readthrough is lethal in *Leishmania* spp. (27).

To explore the conservation of J function among kinetoplastids and avoid indirect effects associated with the use of BrdU and genetic deletions, we utilized DMOG to examine the role of J in regulating RNAP II termination in *L. major* and *T. brucei*. We show that reduction of base J using DMOG in *L. major* resulted in genome-wide transcriptional readthrough at cSSRs and HT sites, without cell death. Strand-specific reverse transcription-polymerase chain reaction (RT-PCR) detection of the nascent transcript confirmed that we are measuring J-dependent defects in transcriptional termination, rather than RNAP II reinitiation events. Complete loss of J in *T. brucei* failed to indicate any defect in termination within cSSRs or HT sites. However, we localized base J at sites prior to the end of a PTU where the loss of J led to upregulated expression of the downstream genes within the same PTU. For one of these sites we show that the gene expression changes occurred at the level of transcription. Therefore, while base J regulates RNAP II termination in both *L. major* and *T. brucei*, it does so to different degrees and at different genomic locations. In *L. major*, J regulates termination at the end of each PTU to prevent the generation of antisense RNAs genome-wide. In contrast, while termination occurs at the end of each PTU in *T. brucei* in a J-independent manner, J-dependent termination within a PTU allows developmentally regulated expression of downstream genes.

## MATERIALS AND METHODS

### Enzymes and chemicals

All restriction enzymes were purchased from New England Biolabs. Prime-It II random primer labeling kit was purchased from Stratagene. ECL (enhanced chemiluminescence) and Hybond-N+ were from Amersham. Goat anti-rabbit HRP (horseradish peroxidase) was purchased from Southern Biotec Inc. All other chemicals were purchased from Sigma Aldrich.

### Parasite cell culture

Bloodstream form *T. brucei* cell line 221a of strain 427 was cultured in HMI-9 medium as described previously (40). *L. major* parasites were grown at 26°C in M199 media supplemented with 10% fetal bovine serum (FBS) as described (41). DMOG treatment of cells was performed by supplementing media with 1-mM DMOG for 5 days in *T. brucei* or at 5-mM for 10 days in *L. major* (1–5 mM for the DMOG titration experiments shown in Figure 3C). BrdU was supplemented into media at 10-μM or 100-μM for 6 days in *L. major*.

**Figure 1.**
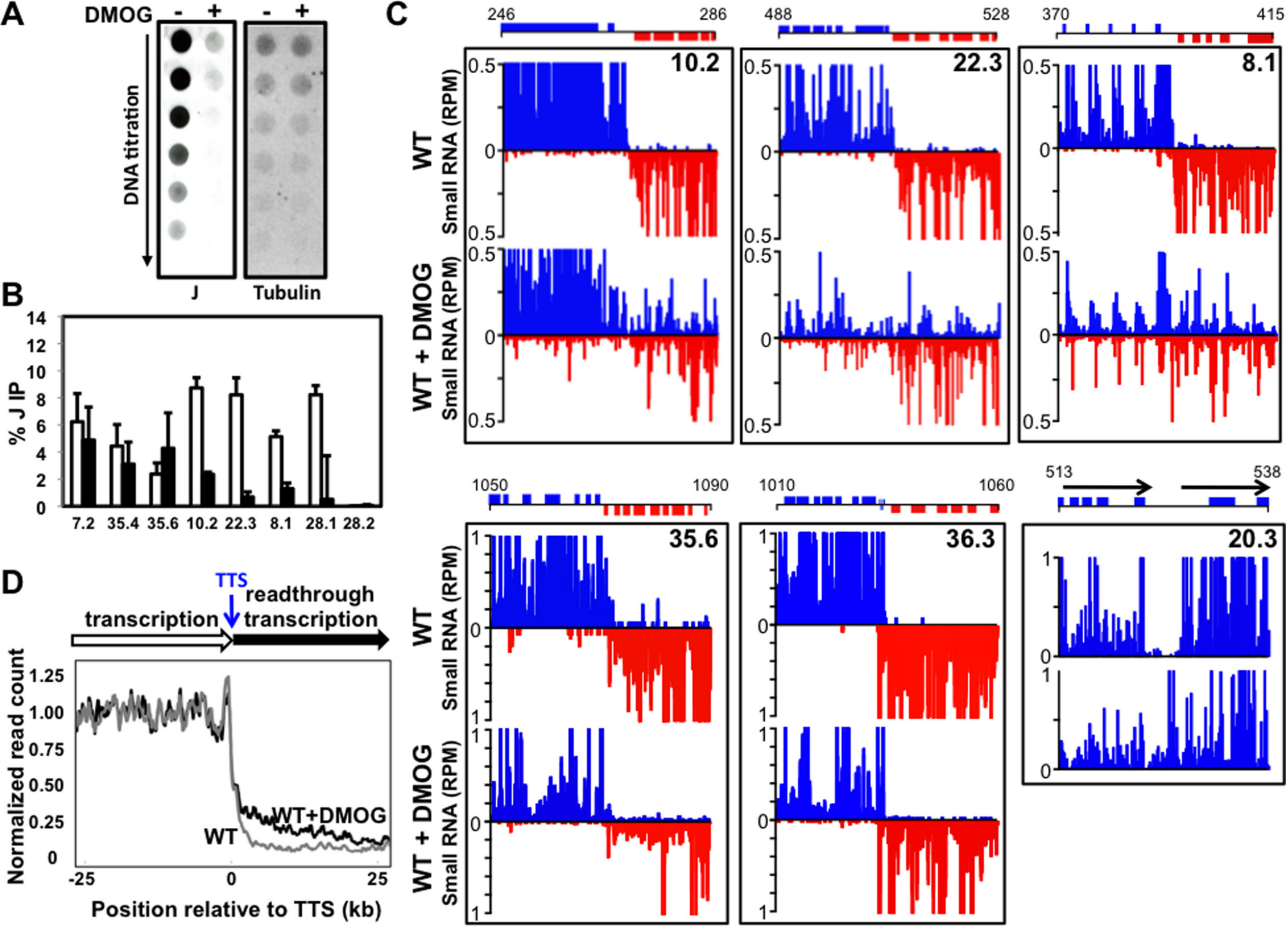
Loss of base J results in readthrough transcription and the production of antisense RNAs in *L. major*. (**A**) Anti-base J dot blot analysis of WT *L. major*. A 2-fold serial dilution of genomic DNA was spotted onto a membrane and incubated with anti-base J antisera. −: DMSO; +: 5-mM DMOG. The membrane was stripped and probed using a radio-labeled beta tubulin probe as a loading control. (**B**) Anti-base J IP qPCR analysis. The average of three independent IPs is plotted as the percent IP relative to the total input material. All IPs were background subtracted using a no antibody control. White bars: DMSO; black bars: DMOG. Seven cSSRs enriched for base J are shown and one previously identified J negative cSSR (28.2) as a negative control (see Supplementary Table S1 for genomic location). Error bars represent the standard deviation. (**C**) Upper panels, small RNA sequencing reads for three cSSRs where J loss led to readthrough transcription are shown. Small RNA reads are plotted as reads per million reads mapped (rpm). Upper graphs: DMSO; lower graphs: DMOG. ORFs and the genomic location (kb) are shown above the graphs. Blue: top strand; red: bottom strand. Lower panels: cSSR 35.6 illustrates a region where J was not reduced by DMOG (see Figure 1B) and there was no readthrough defect; cSSR 36.3 shows a site containing tRNA genes on both DNA strands where J was reduced by DMOG (see Supplementary Figure S1C), but did not result in a readthrough defect; and HT site 20.3 shows a non-cSSR termination site where J loss (see Supplementary Figure S1C) resulted in a termination defect. (**D**) A metaplot summarizing the readthrough defect at cSSRs (*n* = 36, 3 discarded) aligned by their TTS, shown as position 0 on the x-axis. Meta coverage of each sample was normalized by the mean meta coverage of upstream of TTS (see the Materials and Methods section).

**Figure 2.**
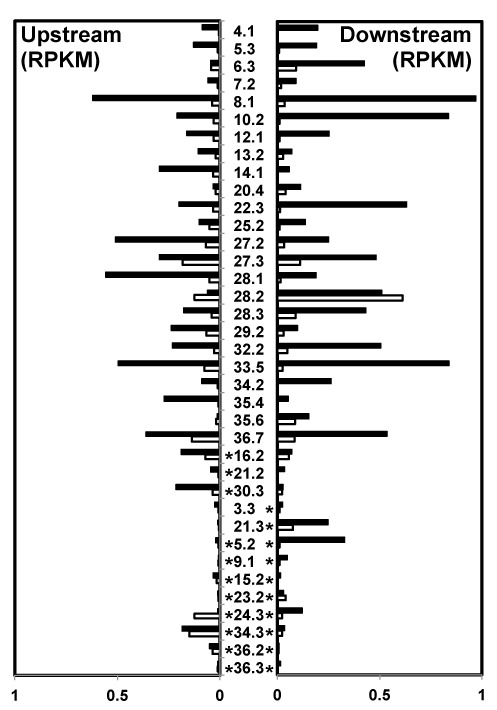
Quantification of readthrough transcription at individual cSSRs in *L. major*. The antisense small RNA reads per kb per million reads mapped (RPKM) within a 5-kb window downstream of the TTS is plotted for each cSSR. cSSRs are labeled in the center. Bars to the left represent readthrough transcription on the bottom strand (readthrough transcription from right to left) and bars to the right represent readthrough transcription on the top strand (readthrough transcription from left to right). White bars: DMSO; black bars: DMOG. cSSRs containing tRNA genes are indicated by an asterisk; cSSRs with tRNAs on the top strand only (* on the right side of the cSSR number), bottom strand only (* on the left side) or both strands. See Supplementary Table S1 for the genomic location of each cSSR. cSSRs 9.2 and 22.2 were excluded here because the downstream PTU was less than 5 kb.

**Figure 3.**
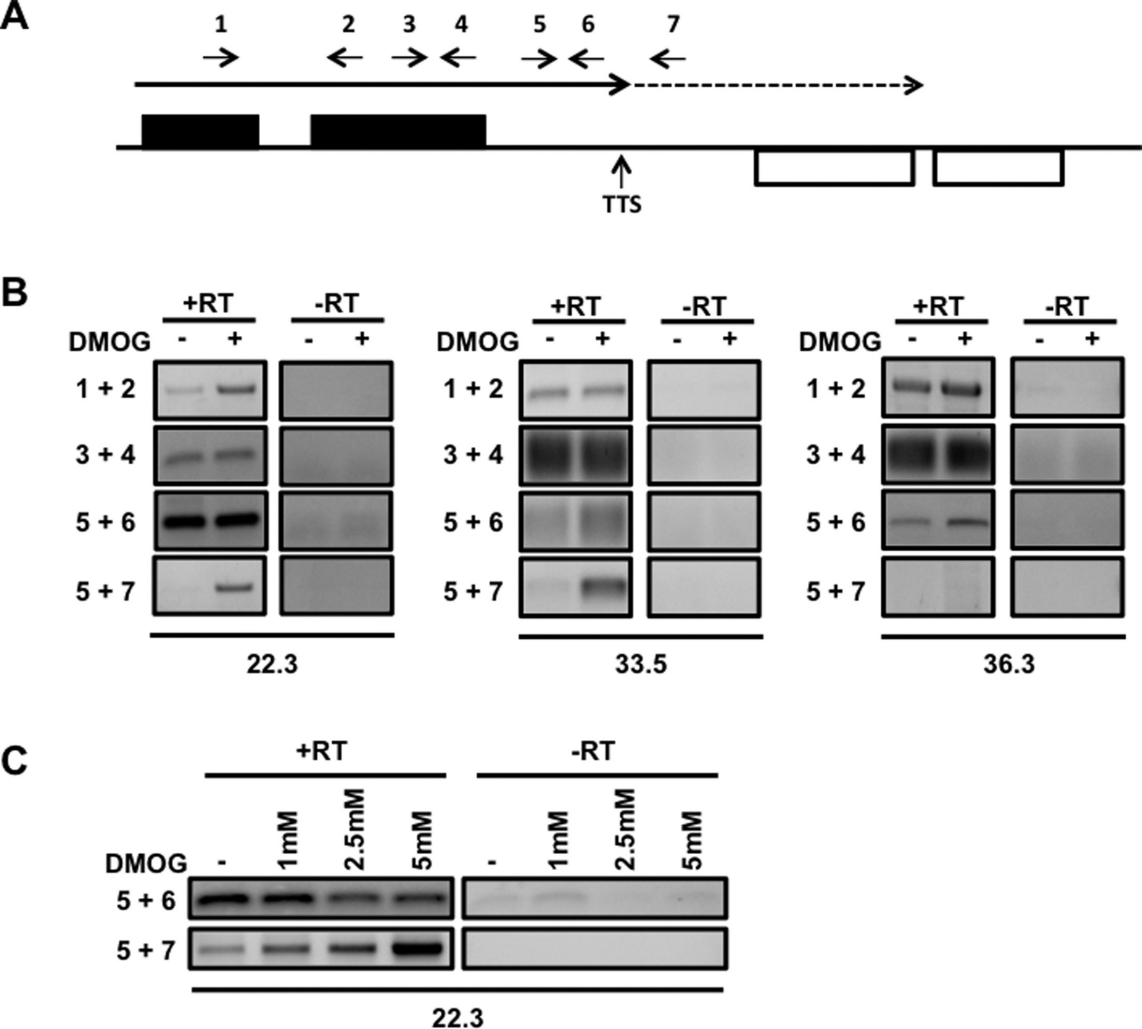
RNAP II fails to terminate following reduction of base J. (**A**) Schematic representation of primer location and direction at a cSSR. The dashed arrow indicates readthrough transcription past the TTS. (**B**) Single-strand RT-PCR analysis. cDNA was synthesized using the reverse primers 2, 4, 6 and 7. PCR was performed using the same reverse primer used to make the cDNA plus a forward primer, as indicated. Three cSSRs were analyzed, two with readthrough upon J loss (22.3 and 33.5) and one without readthrough (36.3), as determined by small RNA-seq analysis. Plus RT and minus RT controls are shown. −: DMSO; +: DMOG (5 mM). (**C**) The amount of readthrough transcription correlates with the extent of J loss. Cells treated with 0, 1, 2.5 and 5-mM DMOG were analyzed by single-strand RT PCR as described above.

### Determination of the genomic level of J

To quantify the genomic J levels, we used the anti-J DNA immunoblot assay as described (24,34) on total genomic DNA, which was isolated as described (42). Briefly, serially diluted genomic DNA was blotted to nitrocellulose followed by incubation with anti-J antisera. Bound antibodies were detected by a secondary goat anti-rabbit antibody conjugated to HRP and visualized by ECL. The membrane was stripped and hybridized with a probe for the beta-tubulin gene to correct for DNA loading.

### The localization of base J

To quantify the levels of base J at specific regions of the *T. brucei* or *L. major* genome, genomic DNA was sonicated and anti J immunoprecipitation (IP) was performed as described (23,24,33,34). Immunoprecipitated J containing DNA was used for quantitative PCR (qPCR) analysis. Input DNA was used as a positive control for qPCR (10% of the IP). Quantification of selected genes was performed on an iCycler with an iQ5 multicolor real-time PCR detection system and iQ5-standard edition version 2.0.148.60623 software (Bio-Rad Laboratories, Hercules, CA, USA). Primer sequences used in the analysis are available upon request. The reaction mixture contained 5 pmol forward and reverse primer, 2x iQ SYBR green super mix (Bio-Rad Laboratories, Hercules, CA, USA), and 2 μl of template DNA. Thermocycling parameters consisted of the following steps: (i) 3 min at 95°C; (ii) 40 cycles of 15 s at 95°C, 30 s at 60°C and 30 s at 72°C; (iii) 1 min at 95°C; (iv) 1 min at 45°C; (v) 101 cycles of 30 s at 45°C, each cycle increasing by 0.5°C, ending at 95°C (melt curve analysis). Standard curves were prepared for each gene using 5-fold dilutions of known quantity (100 ng/μl) of wild-type (WT) DNA. The quantities were calculated using iQ5 optical detection system software.

### Strand-specific RNA-seq library construction

Small RNAs were isolated from *T. brucei* or *L. major* using a Qiagen miRNeasy kit according to the manufacturer's instructions. 5 × 10^7^ cells were used per sample and isolated at the log phase of parasite growth. Total RNA was isolated from log phase *T. brucei* cultures using a Qiagen RNeasy kit according to the manufacturer's instructions. 5 × 10^7^ cells were used for each prep.

All four small RNA-seq libraries were prepared using ∼250 ng small RNA using the TruSeq small RNA kit (Illumina) according to the manufacturer's instructions with the following exception: the PCR amplification was performed for 12–16 cycles using the KAPA HiFi DNA polymerase (Kapabiosystems). The PCR product was purified and concentrated using AMPure XP beads (Beckman Coulter). Quality and concentration of all libraries was determined using a Bioanalyzer 2100 (Agilent) and high-throughput sequencing was performed on a HiSeq2000 (Illumina).

The two full-length RNA-seq libraries were constructed from ∼1 μg of total RNA by Vertis Biotechnology AG. Briefly, total RNA was polyA-enriched using oligo(dT) chromatography and fragmented by ultrasound. Next, first strand cDNA was synthesized using N6 random primers followed by a strand-specific ligation of sequencing adapter to the 3′ and 5′ ends of the first stranded cDNA and PCR amplification of 10–20 cycles depending on the amount of starting material. High-throughput sequencing was performed on a HiSeq2000 (Illumina).

### Mapping of sequence reads, calculations of expression levels and meta-coverage plot

Sequencing reads were mapped to the respective reference genomes (see Supplementary Table S2) using bowtie-2 with default ‘local-sensitive’ mode (43) and further processed using samtools (44). For the small RNA libraries, reads shorter than 18 bp were discarded before mapping. To express the transcript levels for individual genes as shown in Table 1, we determined the number of reads per kilobase per million reads (RPKM) (45). Briefly, we counted the number of reads mapped to all annotated transcriptomic features (e.g. mRNA) on the same strand (i.e. sense) and opposite strand (i.e. antisense). Both the sense and antisense read numbers were normalized by length of the feature (in kilobase) and the total number of reads (in millions) mapped to non-structural RNAs in the corresponding library (i.e. the number of mappable reads excluding rRNA and tRNA reads). For the metaplot of small RNA coverage at transcription termination site (TTS), cSSRs with a TTS located at closer than 50 kb to the edge of the scaffold were discarded. To eliminate positions with abruptly high coverage, positions with coverage greater than 5-fold of the mean sense coverage upstream of the TTS were ignored. The pooled meta-coverage was then smoothed using a sliding window of 500 bp at a step size of 100 bp. The mean sense coverage upstream of a TTS was normalized to 1 within each sample. For the quantification of readthrough transcription at individual cSSRs as shown in Figure 2, the number of antisense small RNA reads per million reads (rpm) was determined within a 5-kb window downstream of the TTS. The antisense small RNA rpm was divided by the window size (5 kb) and expressed as RPKM.

**Table 1.**
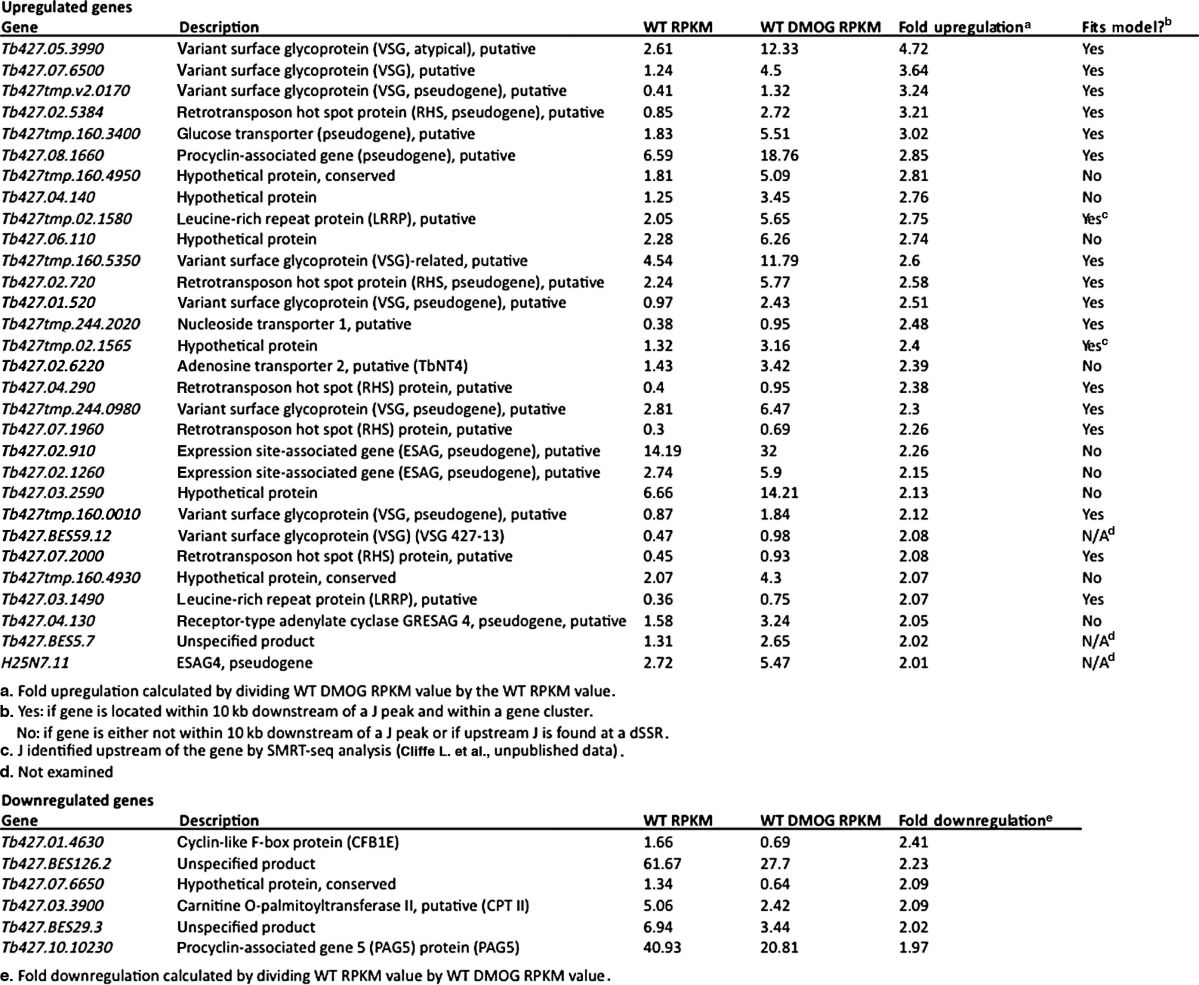
T. brucei gene expression changes following J loss (2-fold or greater)

### Nuclear run on

Wild-type *T. brucei* cultures were grown in the presence of DMSO or 1-mM DMOG for 5 days. Nuclear run on was then performed using 1 × 10^9^ cells as described (26,46). Briefly, parasites were washed twice at room temperature with 20-mM potassium glutamate, 3-mM MgCl2, 150-mM sucrose, 10-μg/ml leupeptin and 1-mM dithiothreitol (transcription buffer) and resuspended in 800 μl of the same buffer. The parasite suspension was chilled on ice for 5 min, and palmitoyl-L-α-lysophosphatidylcholine (Sigma) dissolved in water at 10 mg/ml was added to obtain a final concentration of 500 μg/ml. After 1 min, the parasites were centrifuged, washed once with transcription buffer and resuspended in 200 μl of transcription buffer containing 2-mM adenosine triphosphate (dATP), 1-mM cytosine triphosphate (dCTP), 1-mM guanosine triphosphate (dGTP), 0.6-mg/ml creatine kinase (Roche Molecular Biochemicals), 25-mM creatine phosphate and 100 μCi of [α-32P] uridine triphosphate (UTP) (6000 Ci/mmol). After 20 min at 30 °C, the parasites were centrifuged again; the pellets were lysed with 1 ml of acid phenol/guanidine isothiocyanate; and the labeled RNA was recovered as described above. The labeled RNA was then hybridized in 5× saline/sodium phosphate/ethylenediaminetetraacetic acid, 5× Denhardt's solution, 0.1-mg/ml yeast tRNA and 0.1% sodium dodecyl sulfate (SDS) to dot blots containing 5–10-μg spots of the indicated DNA. For preparation of the dot blots, plasmid DNA containing probes for multi-copy genes and single-copy genes and regions was denatured in 0.6-N NaOH for 15 min at 55 °C, neutralized by adding ammonium acetate to 2 N, and loaded onto nitrocellulose membranes using a minifold filtration apparatus, as previously described (46). Primer sequence used to generate the probes can be provided upon request. The 5.8S (387 bp) and 18S (2188 bp) probes provide a control for RNAP I transcription and the 5S (639 bp) for RNAP III transcription. Tubulin (1300 bp) and SL (500 bp) probes provide control for RNAP II transcription. An empty plasmid (pCR2.1) was used as a negative control. RNAs were incubated with membranes containing probes for 48 h and then subjected to washing with 0.1 x SSC, 0.1% SDS three times at 65°C and then analyzed by phosphorimager. The signal for each probe was background subtracted and normalized to the spliced leader signal.

### Strand-specific RT-PCR analysis of readthrough transcription

Total RNA was isolated using the hot phenol method, as described previously (47). The RNA pellet was resuspended in water and further purified using a Qiagen RNeasy kit according to the manufacturer's instructions. To ensure complete removal of contaminating genomic DNA, the on column DNase I digest step was performed twice. Purified RNA was eluted from the column by water and the concentration was determined using a spectrophotometer. Strand-specific RT-PCR was performed as previously described (48). ThermoScript^TM^ Reverse Transcriptase from Life Technologies was used for cDNA synthesis at 60–65°C. Two micrograms of RNA were used to make cDNA using the reverse primers shown in Figure 3A. PCR was performed using GoTaq DNA Polymerase from Promega. A minus-RT control was used to ensure no contaminating genomic DNA was amplified. Primer sequences used in the analysis are available upon request.

### Reverse transcription quantitative polymerase chain reaction

Total RNA was obtained using Qiagen RNeasy kits according to manufacturer's instructions. First-strand cDNA was synthesized from 1 μg of total RNA using an iScript cDNA synthesis kit (Bio-Rad Laboratories, Hercules, CA, USA) per the manufacturer's instructions. Quantification of selected genes was performed on an iCycler with an iQ5 multicolor real-time PCR detection system (Bio-Rad Laboratories, Hercules, CA, USA). Reaction conditions and thermocycling parameters used were the same as those described above. All transcripts were normalized using 40s rRNA. Briefly, triplicate cDNAs were diluted 1:10 and the 40s cDNA starting quantity (in nanograms) was determined using a standard curve (described above). The ratio of WT:WT DMOG-treated 40s cDNA starting quantity was used to normalize all subsequent transcripts analyzed by RT-qPCR. Primer sequences used in the analysis are available upon request.

## RESULTS

### J regulation of RNAP II termination in *L. major*

#### J regulation of RNAP II termination at cSSRs and HT sites in L. major

We have previously shown that growth of kinetoplastids in DMOG effectively inhibits J synthesis and leads to a large reduction in the total level of base J (29). Wild-type *L. major* grown in medium containing 5-mM DMOG for 10 days resulted in ∼32-fold reduction in the total level of base J (Figure 1A) and little to no growth phenotype (Supplementary Figure S1). In comparison, inhibition of J synthesis by BrdU led to at most an 8-fold reduction in the total level of J and cell death within 6 days (Supplementary Figure S1). Given the more significant reduction in total J observed following DMOG treatment compared to BrdU treatment, and the known toxicity of BrdU to other eukaryotic cells (that lack base J) (37,39,49), it is possible that the BrdU treatment resulted in cell death independent of J loss.

To analyze J levels at specific cSSRs, we performed anti-base J IP followed by qPCR. We found that DMOG reduced chromosome internal J, though J loss was more pronounced at some cSSRs than others (Figure 1B). While both DMOG and BrdU significantly reduced internal J, DMOG inhibition led to greater reductions of base J at telomeres, explaining the enhanced total J loss observed following DMOG treatment (Supplementary Figure S1C). Thus, to reduce J at cSSRs while avoiding genetic deletions and potential toxicity of BrdU, we utilized DMOG to study the specific role of base J in transcription termination.

Similar to the method previously utilized to characterize transcription termination in *L. tarentolae*, we performed high-throughput sequencing on small RNAs from wild-type *L. major* treated with DMOG to identify the effect of J loss at termination sites and evidence of readthrough transcription. This provides a rough picture of the primary transcript map of *L. major* (i.e. mapping TTS), as reflected in small RNA degradation products. As expected, RNAP II transcription generally terminated at a defined location within the cSSR in wild-type cells. As described in *L. tarentolae* (27), the reduction of base J at cSSRs in *L. major* resulted in the production of antisense RNAs corresponding to genes in the opposing PTU presumably due to readthrough transcription (Figure 1C).

A metaplot summarizing the readthrough defect for all cSSRs is shown in Figure 1D and the amount of readthrough at each cSSR is summarized in Figure 2. Readthrough occurred at 30 of the 37 convergent TTSs when J was reduced, though the extent of readthrough varied and was often asymmetric at a given site (Figure 2). Interestingly, the degree of readthrough transcription often correlated with the reduction in J level following DMOG treatment at the cSSRs examined (Figure 1B and C, Figure 2 and Supplementary Figure S1D). As was observed in *L. tarentolae*, readthrough transcription was observed in wild-type cells at the single cSSR (28.2) that normally lacks J (Figures 1B and 2), further supporting the link between J levels and transcription termination. Another variable is the presence of RNA genes transcribed by RNAP III (i.e. tRNAs and snRNAs) in the cSSR (Figure 1B and C and Figure 2). Readthrough transcription was modest at cSSRs containing genes transcribed by RNAP III. For example, DMOG treatment significantly reduced J levels at cSSR 36.3 that contains tRNA genes on both DNA strands, with little to no effect on transcription termination (Figure 1C, Figure 2 and Supplementary Figure S1C). Additionally, the orientation of the RNAP III gene relative to the direction of RNAP II transcription influenced the extent of readthrough observed. At several cSSRs containing an RNAP III gene on the opposing strand, readthrough was modest upon the loss of J. However, at sites where the RNAP III genes are transcribed in the same direction as the adjacent PTU, readthrough was more extensive. For example, see cSSRs 30.3, 21.3 and 5.2 (Figure 2). While there were a few exceptions, these results suggest that RNAP II can bypass an RNAP III transcription unit more readily if the polymerases are traveling in the same direction and that opposing RNAP III transcription allows J-independent termination. Asymmetric readthrough was also seen at cSSRs without RNAP III genes; therefore, additional factors likely contribute to the extent of the readthrough defect observed.

In addition to cSSRs, termination at HT sites was also affected by the loss of base J (Figure 1C and Supplementary Figure S2A). HT regions are enriched for chromatin marks such as base J and acetylated histone H3, as well as the histone variants H3V and H4V and H4ac at similar sites in *T. brucei*, which indicate termination and reinitiation on the same DNA strand (5–7,27). Small RNA-seq analysis further supports transcription termination and reinitiation at HT sites, given the lack of RNAs detected in the region between the upstream and downstream PTUs in wild-type *L. major* (see HT 20.3 Figure 1C and HT 19.4 Supplementary Figure S2A). Upon the loss of J we detected RNAs throughout the HT region, suggesting that J is also involved in regulating RNAP II termination within these sites (Figure 1C, Supplementary Figure S2A and data not shown). Additionally, consistent with a role of RNAP III genes in preventing readthrough transcription, little readthrough was observed at an HT site containing tRNA genes on both DNA strands (Supplementary Figure S2B).

#### Detection of nascent RNA generated by RNAP II readthrough

As previously discussed (27), the generation of small RNAs downstream of putative TTSs following the loss of base J is presumably due to readthrough transcription and continued elongation of the RNAP II machinery rather than transcription reinitiation events. To better define the observed defect as transcriptional readthrough, we utilized strand-specific RT-PCR to directly detect the extension of nascent RNA transcripts. Figure 3A illustrates the orientation and position of primers relative to the TTS. Primers 1 and 2 spanned two adjacent open reading frames (ORFs) and ensured our ability to analyze unprocessed, nascent RNA transcripts. Primers 6 and 7 were positioned upstream and downstream, respectively, of the TTS, as defined by small RNA-seq of wild-type cells. Consistent with the TTS mapping, we detected a nascent RNA transcript upstream of the TTS (using primers 5 and 6), but not the one that spanned the TTS (using primers 5 and 7) in wild-type *L. major* (Figure 3B). Consistent with J-dependent transcriptional readthrough, for each of the cSSRs tested we detected a nascent RNA transcript upstream of the TTS in both wild-type and DMOG-treated *L. major*; however, a readthrough product that spanned the TTS was present only when J levels were reduced. Any possible transcriptional reinitiation events occurring downstream of the TTS would not generate an RNA molecule that spans the TTS. As an additional control, a readthrough product was not observed in wild-type or DMOG-treated cells at a (tRNA containing, 36.3) site that lacked a detectable readthrough defect by small RNA-seq. A readthrough product was also not observed at a site (35.6) where DMOG failed to significantly reduce J and no readthrough defect was detected by small RNA-seq (Supplementary Figure S3). We conclude that the small RNA-seq analysis accurately measures termination defects where base J regulates RNAP II termination at the end of PTUs to prevent transcriptional readthrough in *L. major*. Furthermore, consistent with a direct relationship between the strength of RNAP II termination and level of base J, we detected increased amounts of readthrough product with increased concentrations of DMOG (1–5 mM) (Figure 3C). We have previously demonstrated that this DMOG titration leads to quantitative reduction in base J in *L. major* (29). Overall these results suggest that J functions to regulate termination throughout the genome of *L. major*, but that this significant readthrough termination defect and generation of antisense RNAs is not lethal to the cell.

### J regulation of RNAP II termination in *T. brucei*

#### J regulation of RNAP II termination at cSSRs and HT sites in T. brucei

**Figure 4.**
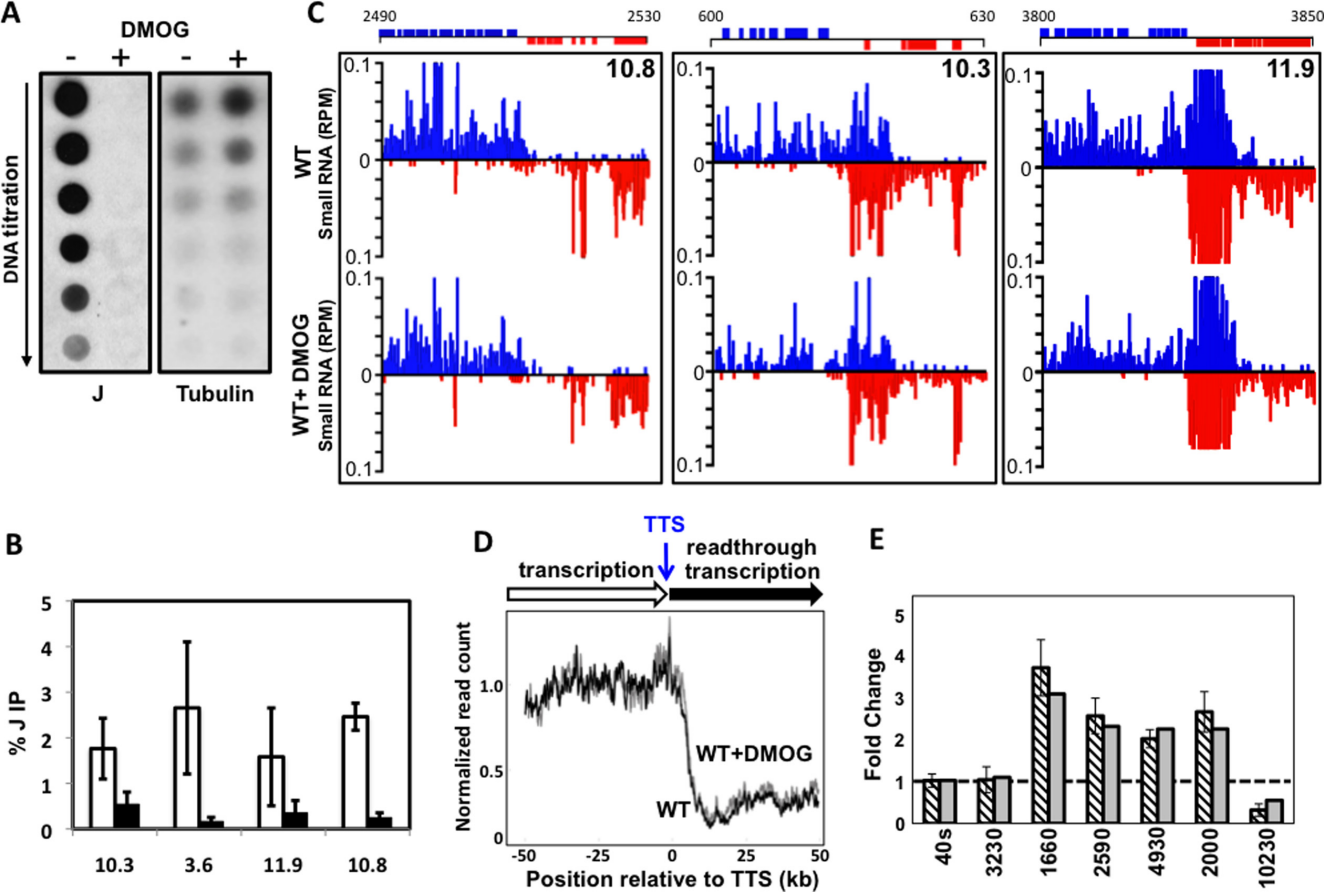
Loss of base J does not lead to readthrough transcription in *T. brucei* at cSSRs. (**A**) Anti-base J dot blot analysis of WT *T. brucei* was performed as detailed for Figure 1A, but *T. brucei* genomic DNA was isolated from cells treated with either 1-mM DMOG or DMSO for 5 days. (**B**) Anti-base J IP qPCR analysis. Same as shown in Figure 1B, where white bars show % IP for DMSO and black bars show % J IP from DMOG-treated *T. brucei* DNA. Four cSSRs enriched for base J are shown. Error bars represent the standard deviation. (**C**) Small RNA-seq analysis of three cSSRs (10.8, 10.3 and 11.9) is shown with reads plotted as reads per million reads mapped (rpm). Top graphs: DMSO-treated WT; bottom graphs: DMOG-treated WT. Reads mapped to the top strand are shown in blue and reads mapped to the bottom strand in red. ORFs and their chromosomal location in kb are shown above. (**D**) A metaplot summarizing the readthrough defect at cSSRs (*n* = 87, 11 discarded) aligned by their TTS, shown as position 0 on the x-axis. Meta coverage of each sample was normalized by the mean meta coverage of upstream of TTS (see the Materials and Methods section). (**E**) Confirmation of total RNA-seq transcript changes in *T. brucei* by RT qPCR. Fold change in transcript abundance, with DMSO-treated WT set to 1. Striped bars: fold change based on RT qPCR analysis of DMOG-treated WT *T. brucei*; gray bars: fold change in the RPKM of DMOG-treated WT *T. brucei* based on total RNA-seq analysis. For RT qPCR analysis, transcripts were normalized against 40s rRNA. Transcripts analyzed included 3230 (Tb427.07.3230), which did not change in abundance after DMOG treatment; 1660 (Tb427.08.1660), 2590 (Tb427.03.2590), 4930 (Tb427tmp.160.4930) and 2000 (Tb427.07.2000), which were increased by at least 2-fold after DMOG treatment; and 10230 (Tb427.10.10230), which was decreased about 2-fold following DMOG treatment. Error bars represent the standard deviation of three independent biological replicates.

The termination defects observed in *Leishmania* spp. prompted us to examine *T. brucei* for similar transcription termination defects upon the loss of base J. Base J is not essential in *T. brucei*, as both JBP enzymes can be knocked out without obvious phenotypic effects (34). Consistent with this, wild-type *T. brucei* treated with 1-mM DMOG reduced global J levels beyond the limits of detection by anti-J dot blot (Figure 4A), with no significant growth defect (Supplementary Figure S4). Consistent with total J levels, all cSSRs examined had significantly reduced levels of J following DMOG treatment (Figure 4B).

To measure readthrough defects, we performed small RNA-seq analysis as described above. In contrast to *Leishmania* spp., we found no evidence of termination defects in *T. brucei* at cSSRs or at HT regions following J loss. Antisense small RNAs, indicative of readthrough transcription at cSSRs into the downstream PTU, were not increased following the loss of base J (Figure 4C). Similarly, no significant changes in small RNAs corresponding to readthrough transcription at HT sites were detected (Supplementary Figure S5). A metaplot summarizing the lack of readthrough defects at the ∼100 cSSRs is shown in Figure 4D. During this analysis, unique to *T. brucei*, we identified peaks of sense and antisense small RNAs that mapped near and within some cSSRs of wild-type parasites in the absence of DMOG (Figure 4C; 10.3 and 11.9). These presumably represent the previously identified Argonaute-associated siRNAs derived from cSSRs (50). Regardless of their source, the level of (siRNA-like) sense/anti-sense small RNAs that map to particular cSSRs was not changed by the loss of J (Figure 4C and D). Thus, in contrast to the readthrough transcription observed in *Leishmania* spp., the loss of J did not affect RNAP II termination at cSSRs or HT regions in *T. brucei*.

#### J regulation of RNAP II termination within PTUs in T. brucei

To further explore the role of J in regulating RNAP II transcription in *T. brucei*, we investigated the effect of J loss on transcript abundance using total RNA-seq. In contrast to the global changes detected in *T. cruzi* and *L. tarentolae* following decreased levels of base J (26,27), we identified very limited gene expression changes in *T. brucei* cells incubated with DMOG (Table 1). RNA-seq indicated only 36 transcripts were changed more than 2-fold following the loss of base J, the majority of which (30 transcripts) had increased expression. It is interesting to note that the majority of genes with increased mRNA levels are annotated as VSGs, RHS proteins, ESAGs and pseudogenes and are lowly expressed (or silent) in wild-type *T. brucei*. We confirmed many of these changes by RT qPCR (Figure 4E) and removal of DMOG restored base J synthesis and wild-type expression levels (Supplementary Figure S6A). Similar changes in transcript abundance were also observed in the J null cell line (JBP1/JBP2 KO) indicating the expression changes were not due to indirect effects of the DMOG treatment (Supplementary Figure S6B). While total RNA-seq revealed six genes with at least a 2-fold reduction in mRNA upon the loss of base J (Table 1), we were only able to confirm the transcript changes for one out of three transcripts analyzed by RT qPCR (Figure 4E and data not shown). We have not followed up further on the few genes that may have decreased expression following loss of J.

**Figure 5.**
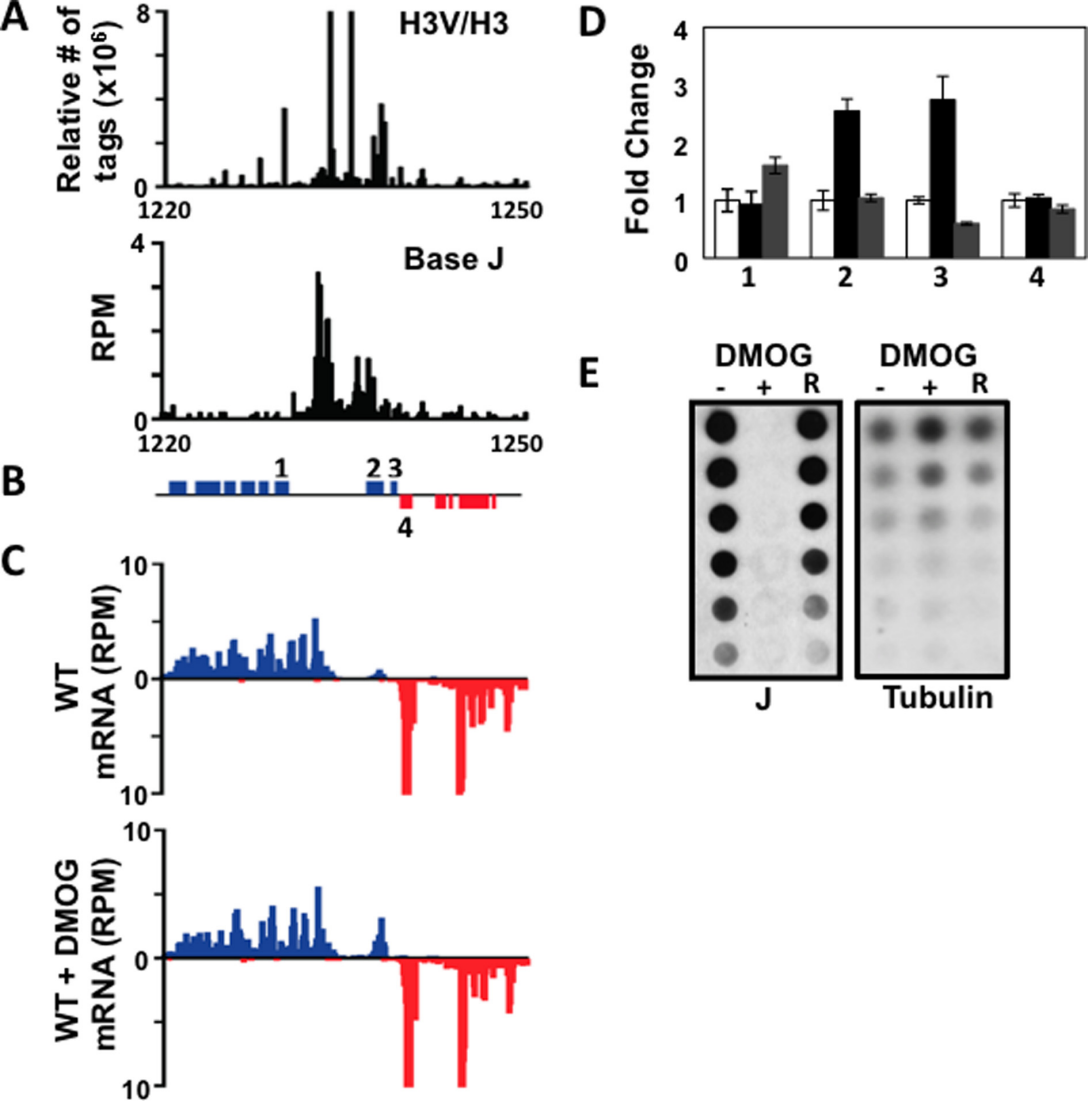
Base J regulates gene expression at the level of RNAP II transcription. A region on chromosome 5 is shown where base J regulates RNAP II transcription. (**A**) Base J and H3V co-localize at sites of RNAP II termination. H3V ChIP-seq reads are plotted as the relative number of sequence tags normalized to H3 ChIP-seq reads, as previously described (5) and base J IP-seq reads are plotted as reads per million mapped reads (rpm). (**B**) ORFs are shown with the top strand in blue and the bottom strand in red. Genes analyzed by RT qPCR in (**D**) are labeled 1–4; 1: Tb427.05.3980 (hypothetical protein, conserved); 2: Tb427.05.3990 (variant surface glycoprotein, atypical, putative); 3: Tb427.05.4000 (hypothetical protein); and 4: Tb427.05.4010 (hypothetical protein). (**C**) Total RNA-seq reads plotted as rpm. Mapped reads from DMSO-treated WT *T. brucei* are shown above and those from DMOG-treated WT *T. brucei* are shown below. Reads that mapped to the top strand are shown in blue and reads that mapped to the bottom strand in red. (D) RT qPCR confirmation of transcript changes. The transcript fold change is shown for the four genes (1–4) shown in (B). White bars: DMSO-treated WT, set to 1; black bars: DMOG-treated WT; and gray bars: J rescue, where cells were grown for 10 days in medium without DMOG, allowing J to be re-synthesized. Error bars represent the standard deviation of three independent biological replicates. (**E**) Anti-base J dot blot analysis. −: DMSO-treated WT; +: DMOG-treated WT; and R: J rescue.

**Figure 6.**
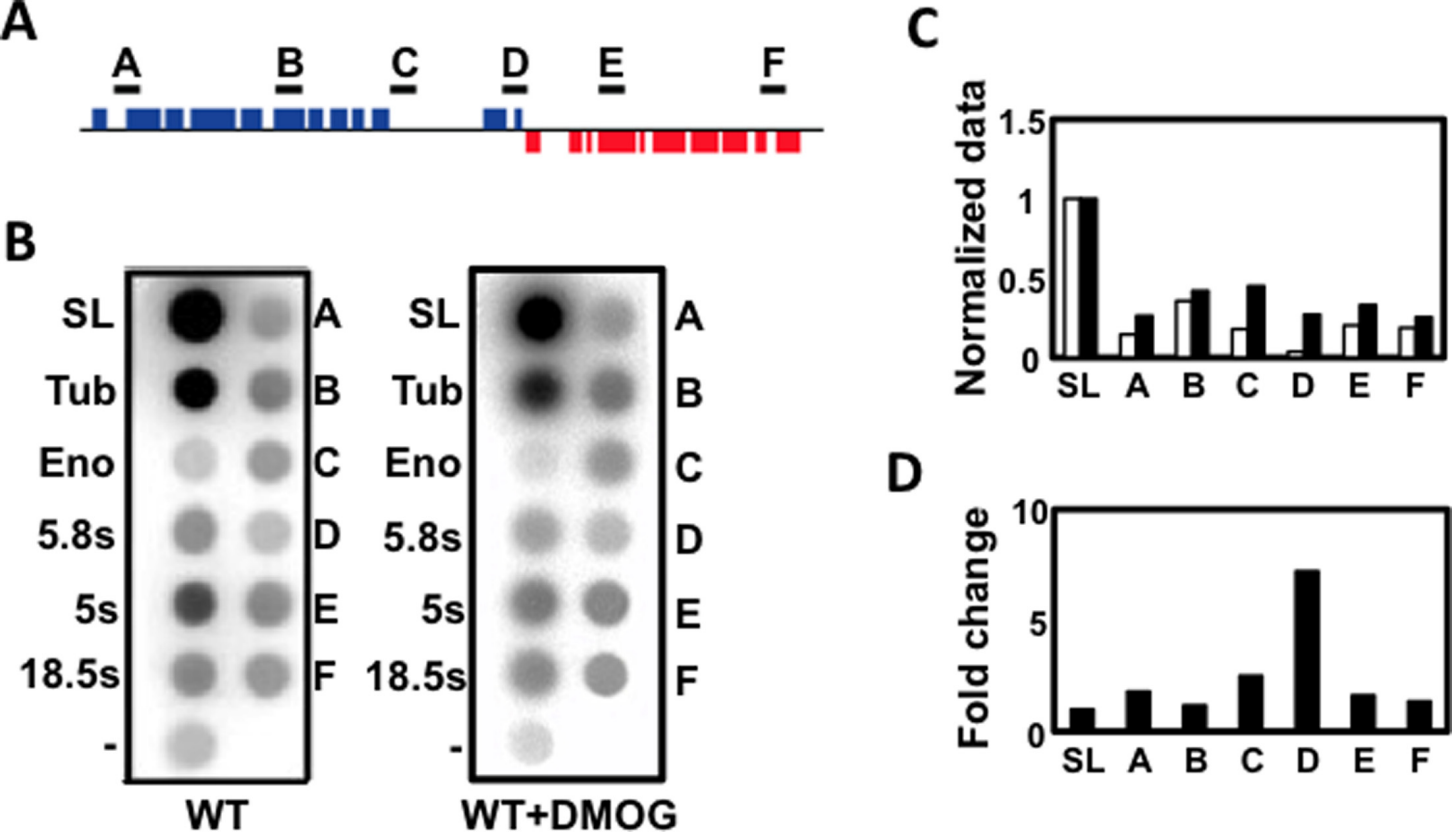
Nuclear run-on analysis of the region shown in Figure 5. (**A**) The location of probes A–F is shown. (**B**) Results from a representative experiment are shown, with DMSO-treated WT on the left and DMOG-treated WT on the right. The probes used include SL: spliced leader; Tub: tubulin; Eno: enolase; 5.8S: 5.8S rRNA; 5S: 5S rRNA; 18.5S: 18.5S rRNA; −: empty pCR2.1 vector; and probes A–F illustrated above. The radioactive signal was measured using a phosphorimager and normalized using the SL signal. The empty pCR2.1 vector was used to subtract background signal. (**C**) The normalized signal—white bars: DMSO; black bars: DMOG. (**D**) Fold change in the DMOG-treated WT signal, with the DMSO-treated WT signal set to 1.

Interestingly, 18 out of the 27 upregulated genes (67%) are located downstream of a J peak within a gene cluster, suggesting that base J may facilitate termination prior to the end of a gene cluster and attenuate the transcription of downstream genes. Consistent with RNAP II termination prior to the end of a PTU, H3V is also enriched upstream of many of the upregulated genes (5). An example is shown in Figure 5, where a peak of base J and H3V is found upstream of the last two genes in a PTU. Both of the downstream genes are lowly expressed in the presence of base J, but increased upon the loss of J (Figure 5A–C) (for additional examples see Supplementary Figure S7). As described above, removal of DMOG restored base J synthesis and wild-type expression levels (Figure 5D and E). To examine whether the upregulation was due to increased transcription, as opposed to post-transcriptional mechanisms, we performed nuclear run-ons. Consistent with transcription termination occurring prior to the last two genes in this gene cluster, the signal from elongating RNAP is lost between the region covered by probes C and D in WT cells (Figure 6). Termination in this region is further supported by the RNA-seq data and localization of base J and H3V (Figure 5A–C). Upon the loss of base J, we found increased transcription of the region downstream of the peak of J (Figure 6 and Supplementary Figure S8), suggesting that increases in mRNA abundance for these two genes occurred at the level of transcription. Further support for readthrough transcription at this site is provided by single-strand RT-PCR analysis (Supplementary Figure S9). Following the loss of base J we detect an increase in a nascent transcript that extends downstream of the proposed termination site. Overall these results indicate that while base J may not regulate RNAP II termination in *T. brucei* at previously defined sites at cSSRs and HT regions, it may attenuate transcription elongation within specific PTUs and enable regulated expression of downstream genes.

## DISCUSSION

We have clearly demonstrated an important function for base J in the regulation of RNAP II termination in *L. major*, as was observed in *L. tarentolae*. Whereas previous analyses utilized a JBP2 KO treated with BrdU, the use of DMOG as an alternative method to reduce J levels in wild-type cells strongly suggests the epigenetic modification itself regulates RNAP II termination and that termination defects were not due to the loss of JBP2 protein or an indirect effect of BrdU or DMOG. We also demonstrate that J functions to regulate termination at HT sites in *L. major* in addition to cSSRs. Finally, we confirmed the termination defects identified by small RNA-seq as readthrough transcription using strand-specific RT-PCR. These findings suggest antisense RNAs were produced as a result of readthrough transcription, though we cannot exclude the possibility that some RNAP II reinitiation contributed to the production of additional antisense RNAs.

The lethality of BrdU treatment in *L. tarentolae* JBP2 KO cells led to the conclusion that readthrough transcription and subsequent generation of antisense RNAs were responsible for cell death (27). However, BrdU is known to be toxic to other eukaryotic cells (that lack base J) and it has been shown that BrdU incorporation into DNA affects nucleosome positioning on plasmids in yeast cells (38) and gene expression in mammalian cells (51,52). We found that drastic reduction of base J in wild-type *L. major* via DMOG caused global readthrough transcription without a significant effect on cell growth. While we were not able to directly compare the extent of readthrough between the two studies or clarify why the JBP2 KO is hypersensitive to BrdU, our findings suggest *Leishmania* spp. can tolerate the production of antisense RNAs and implicate BrdU toxicity as a potential cause of cell death. Despite the large decrease in total J, and comparable reduction in J by BrdU and DMOG at several of the cSSRs analyzed, DMOG treatment did not reduce J at all cSSRs. Importantly, at the three cSSRs analyzed that had minimal loss in base J, less readthrough transcription was observed compared to the cSSRs analyzed with greater J loss, strengthening the correlation between base J and termination defects. However, the lack of readthrough at some cSSRs could have also influenced cell viability. To address this, as well as the possibility that further J loss in a JBP2 KO cell line influences cell viability, studies more directly comparing BrdU and DMOG treatment in a JBP2 KO cell line are underway.

In addition to base J, RNAP III transcribed genes also appear to facilitate RNAP II termination in an orientation-dependent fashion. One possibility is that RNAP II and III complexes transcribing in the opposite direction of each other collide, thus preventing further transcription, whereas RNAP complexes transcribing in the same direction are able to bypass each other. Such RNAP complex collision, and subsequent effects on transcription, has been observed in yeast (53). Further work in wild-type and DMOG-treated *L. major* will allow us to explore the mechanism by which RNAP III transcribed genes facilitate J-independent RNAP II termination.

In contrast to *Leishmania* spp., the loss of base J did not affect RNAP II termination at the majority of TTSs in *T. brucei*. However, we found that J attenuated RNAP II transcription at some specific genomic loci to regulate the expression of downstream genes. A major difference observed between *Leishmania* spp. and *T. brucei* was the presence of small dsRNA peaks within cSSRs, which we assume represent siRNAs, as previously documented (50,54). *T. brucei* has a functional RNAi pathway (55), though the function of cSSR-derived siRNAs and whether they play a role in regulating RNAP II termination remains unknown. Little is known about how the siRNAs are generated from cSSRs, such as which RNAP is responsible for their transcription. The lack of chromatin modifications associated with transcription initiation at siRNA generating loci is suggestive of continued RNAP II transcription from the upstream PTU. Nonetheless, base J did not affect the production of cSSR-derived siRNAs, nor did the presence or absence of siRNAs within a cSSR influence whether readthrough transcription occurred upon J loss. Furthermore, significant increases in cSSR-derived siRNAs in *T. brucei*, induced by the deletion of a chromatin-bound factor, were not associated with changes in RNAP II termination (Reynolds D. *et al*., unpublished data).

Although small RNA-seq analysis in *T. brucei* failed to detect any termination defect, total RNA-seq analysis identified several sites where J appeared to promote transcription termination prior to the end of a polycistronic gene cluster. The loss of base J within a PTU resulted in increased transcript abundance of genes downstream of the J peak. The majority of these sites are devoid of chromatin modifications associated with transcription initiation, such as histone variants H2AZ, H2BV or histone acetylation, but are enriched for histone variants H3V and H4V, which are associated with transcription termination in *T. brucei* (5). Nuclear run-on analysis supports the conclusion that the loss of base J within a gene cluster led to readthrough transcription and increased mRNA abundance of downstream genes. Several of the upregulated genes were not downstream of base J however, and therefore we cannot exclude the possibility that some of the observed changes in transcript abundance were due to increased RNAP II initiation or altered post-transcriptional regulatory processes. These genes are listed in Table 1 as genes that do not fit our model of readthrough transcription upon J loss. Several other genes are listed as not fitting with our model because the upregulated gene is downstream of a J peak that is associated with a dSSR, thus precluding readthrough transcription as an obvious cause of increased transcription.

Many of the upregulated genes were internal VSGs, ESAGs, RHS proteins and pseudogenes that are normally lowly expressed in wild-type *T. brucei*. While only modest upregulation was observed for most of the genes following J loss, these findings are consistent with a bloodstream stage-specific function of base J in the attenuation of RNAP II transcription and the promotion of transcription termination. The functional significance of this J-dependent regulation during trypanosome bloodstream infections is currently unclear.

The arrangement of functionally unrelated genes within the same PTU seemingly precludes regulated transcription as a mechanism to control gene expression. This work however suggests that placement of genes downstream of base J within a PTU can effectively reduce their expression in *T. brucei*. It is not currently clear whether *Leishmania* spp. regulate gene expression similarly; however, at least one potential site on chromosome 3 in *Leishmania* spp. can be found where base J is upstream of an ORF prior to the end of a gene cluster (27), raising the possibility that other kinetoplastids utilize J to regulate gene expression within a PTU. These findings are consistent with work by Kelly *et al.* that has indicated that the location of a gene within a PTU can impact its expression (56). Thus, regulated expression of genes within PTUs can be achieved through their spatial organization and position relative to base J.

Based on a model in which base J attenuates RNAP II elongation in *T. brucei*, we would expect not only increases in the transcript abundance of downstream genes but also an increase in the small RNAs, reflecting transcription of these regions upon the loss of base J. Upon J loss however, we found no significant increase in the small RNAs in the region downstream of the peak of J (data not shown). Our nuclear run-on analysis supports the conclusion that increases in transcript abundance were due to increased transcription; therefore, it is possible that small RNA-seq analysis does not provide a complete picture of the primary transcriptome in *T. brucei*. It is plausible that the presence of the RNAi machinery affects the pool of small RNAs detectable by the deep sequencing approach used here, which requires a 5′-P and a 3′-OH. Consistent with this possibility, in *Leishmania braziliensis*, which contains a functional RNAi pathway, small RNAs indicative of readthrough are not observed at the single cSSR that lacks base J, in contrast to other RNAi negative *Leishmania* spp. [(27) and demonstrated here]. Whether this is because the RNAi machinery differently processes the small RNAs or because readthrough does not occur at this region is not known.

The non-essential role of base J in *T. brucei* is consistent with the developmentally regulated synthesis of base J and absence of the modification in the procyclic stage of the parasite (24). It is possible that alternative J-independent mechanisms evolved to regulate RNAP II termination during the procyclic life-stage of the parasite within the tsetse fly. Additionally, unlike *Leishmania* spp., where base J is found in all but one cSSR (27), J is not found at all cSSRs in *T. brucei* (18). Thus, base J plays an important function in regulating RNAP II termination genome-wide in *Leishmania* spp., but in *T. brucei* J has a more specialized function attenuating transcription and regulating gene expression at specific genomic loci. Additional experiments are underway to explore the mechanism through which J promotes RNAP II termination, specifically investigating whether the modification directly inhibits RNAP II elongation.

## ACCESSION NUMBER

All sequencing data discussed in this publication have been deposited in NCBI's Gene Expression Omnibus and are accessible through GEO Series accession number GSE57621.

## SUPPLEMENTARY DATA

Supplementary Data are available at NAR Online.

SUPPLEMENTARY DATA
