# Supplementary material for: Regulation of transcription termination by glucosylated hydroxymethyluracil, base J, in Leishmania major and Trypanosoma brucei
Source: Nucleic Acids Res. 2014 Aug 7;42(15):9717–29. doi: 10.1093/nar/gku714 (PMC4150806; doi:10.1093/nar/gku714)
Supplement: SUPPLEMENTARY DATA [file supp_42_15_9717__index.html]

Regulation of transcription termination by glucosylated hydroxymethyluracil, base J, in Leishmania major and Trypanosoma brucei — Regulation of transcription termination by glucosylated hydroxymethyluracil, base J, in Leishmania major and Trypanosoma brucei — SUPPLEMENTARY DATA 

# Regulation of transcription termination by glucosylated hydroxymethyluracil, base J, in *Leishmania major* and *Trypanosoma brucei*

## SUPPLEMENTARY DATA

**Files in this Data Supplement:**

- SUPPLEMENTARY DATA
