# Supplementary material for: Regulation of transcription termination by glucosylated hydroxymethyluracil, base J, in Leishmania major and Trypanosoma brucei
Source: Nucleic Acids Res. 2014 Aug 7;42(15):9717–29. doi: 10.1093/nar/gku714 (PMC4150806; doi:10.1093/nar/gku714)
Supplement: SUPPLEMENTARY DATA [file supp_gku714_nar-01401-v-2014-File009.pdf]

Supplemental Figure 1

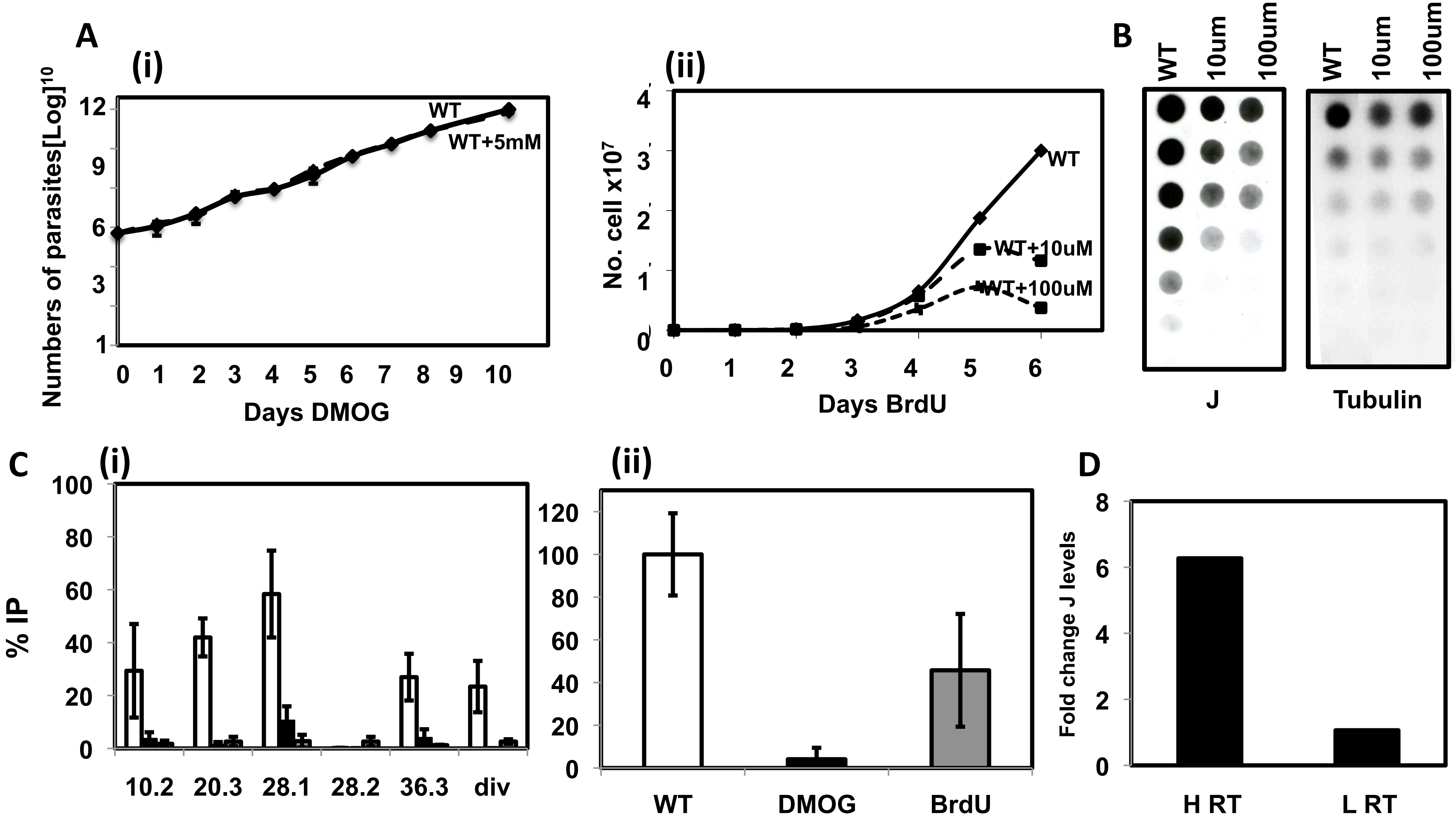

Supplemental Figure 1. DMOG reduces base J and does not result in cell death in *L. major*.

(A) Growth curves of DMSO (WT) and 5mM DMOG (WT+5mM) treated WT *L. major* (i) and 10µM and 100µM BrdU treated WT *L. major* (ii)

(B) Anti-base J dot blot analysis of total J levels in WT and BrdU treated cells, as indicated. DNA was isolated from cells on day 6.

(C) J IP qPCR analysis. White bars, DMSO; black bars, DMOG treated cells; and grey bars, BrdU treated cells. (i) cSSRs analyzed are indicated below the graph. Div indicates a divergent strand switch region (RNAP II initiation site). (ii) qPCR analysis of the telomeric repeats. Error bars represent the standard deviation of three independent IPs.

(D) The average fold reduction in J levels at cSSRs with high readthrough (HRT) and cSSRs with low readthrough (LRT). HRT cSSRs include 10.2, 22.3, 8.1, and 28.1. LRT cSSRs include 7.2, 35.4, and 35.6.

Supplemental Figure 2

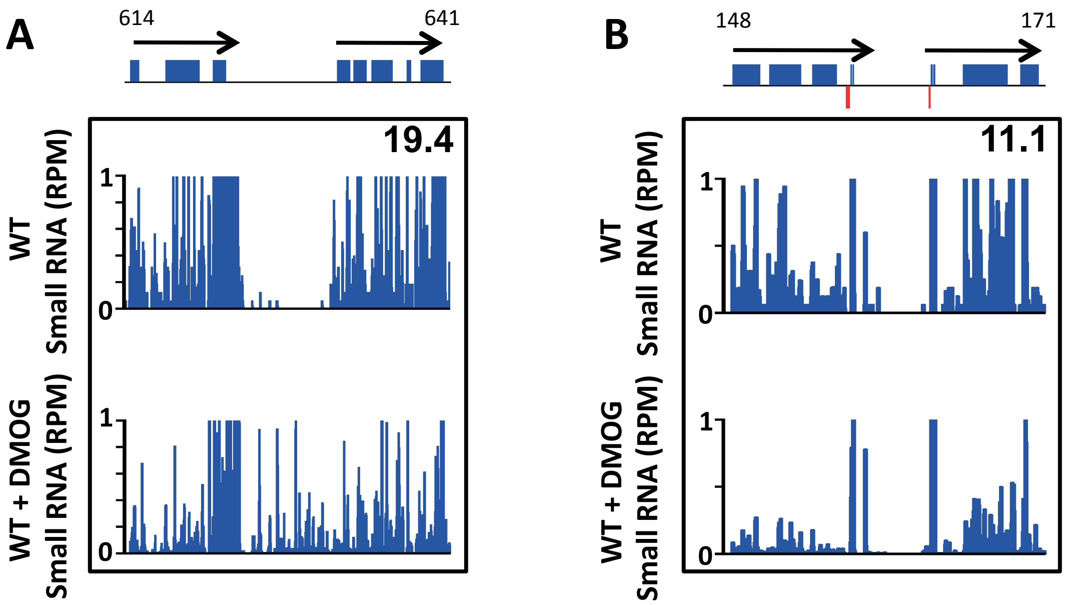

Supplemental Figure 2. RNAP III genes at HT sites prevent RNAP II transcriptional readthrough upon J reduction in *L. major*.

(A) A HT site on chromosome 19 from position 614-641kb is shown. Top, the location of ORFs. Genes on the top strand are shown in blue (no annotated genes present on the bottom strand). Arrows indicate the direction of RNAP II transcription. Small RNA-seq reads were mapped and are shown as reads per million reads mapped (rpm) for both DMSO treated WT *L. major* and DMOG treated WT *L. major*. Only reads mapped to the top strand are shown.

(B) Same as (A), but a HT site containing tRNA genes on chromosome 11 from position 148-171kb is shown. tRNA genes are indicated by thin lines in the center. Genes on the top strand are shown in blue, bottom strand in red.

Supplemental Figure 3

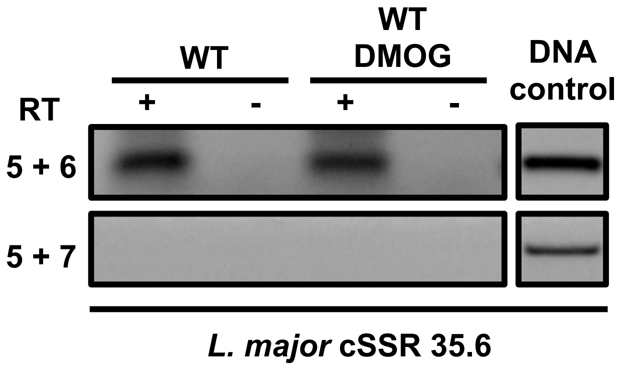

Supplemental Figure 3. Analysis of readthrough transcription in *L. major*.
L. major cSSR 35.6, where DMOG treatment did not significantly reduce base J, was analyzed by single-strand RT PCR. Primers correspond to the schematic shown in Figure 3A. Plus RT and minus RT controls are shown. WT cells were treated with DMSO only. Genomic DNA was used as a PCR positive control.

Supplemental Figure 4

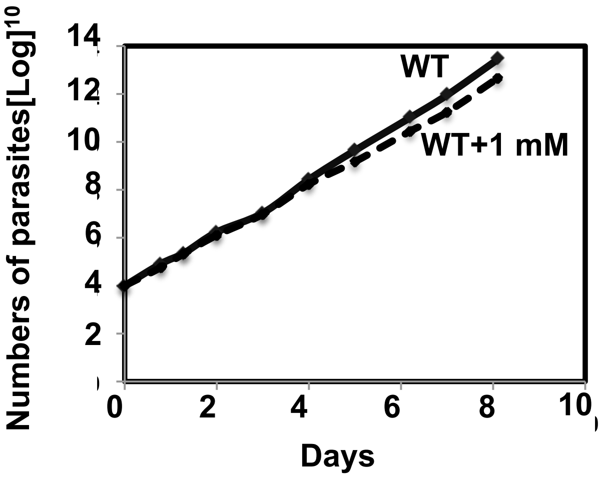

Supplemental Figure 4. WT *T. brucei* treated with 1mM DMOG (WT+1mM) does not result in a growth phenotype. Cell number is plotted on a log_{10} scale. WT DMSO and WT+1mM DMOG are plotted.

Supplemental Figure 5

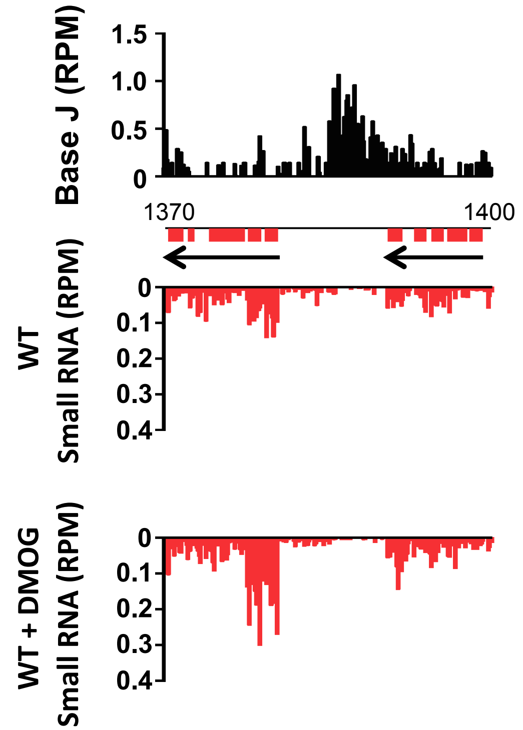

Supplemental Figure 5. Loss of base J from head-tail sites in *T. brucei* does not lead to readthrough transcription. A HT site on chromosome 9 from position 1370-1400kb is shown. Mapped reads from base J IP-seq are shown at the top, plotted as reads per million reads mapped (rpm). ORFs are illustrated below. Small RNA-seq reads from DMSO treated WT and DMOG treated WT are plotted as rpm. Only reads mapped to the bottom strand are shown.

Supplemental Figure 6

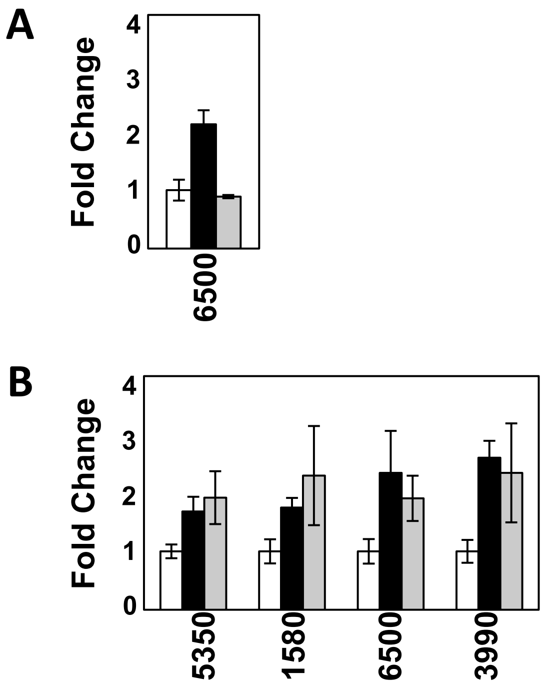

Supplemental Figure 6. Transcript increases in DMOG treated WT *T. brucei* can be rescued and are also found increased in JBP nulls (JBP1 and JBP2 KO).

(A RT qPCR analysis of transcripts from Tb427.07.6500, 6500. White bar, DMSO treated; black bar, DMOG. The grey bar represents a J rescue, where DMOG treated cells were grown in the absence of DMOG for 10 days. Error bars represent the standard deviation of three independent biological replicates.

(B)) RT qPCR analysis of genes found up-regulated by total RNA-seq following J loss. White bars, DMSO treated WT; black bars, DMOG treated WT; grey bars, JBP Null. DMSO treated WT was set to 1. Error bars represent the standard deviation of three independent biological replicates. Genes analyzed were 5350, Tb427tmp.160.5350; 1580, Tb427tmp. 02.1580; 6500, Tb427.07.6500; and 3990, Tb427.05.3990.

Supplemental Figure 7

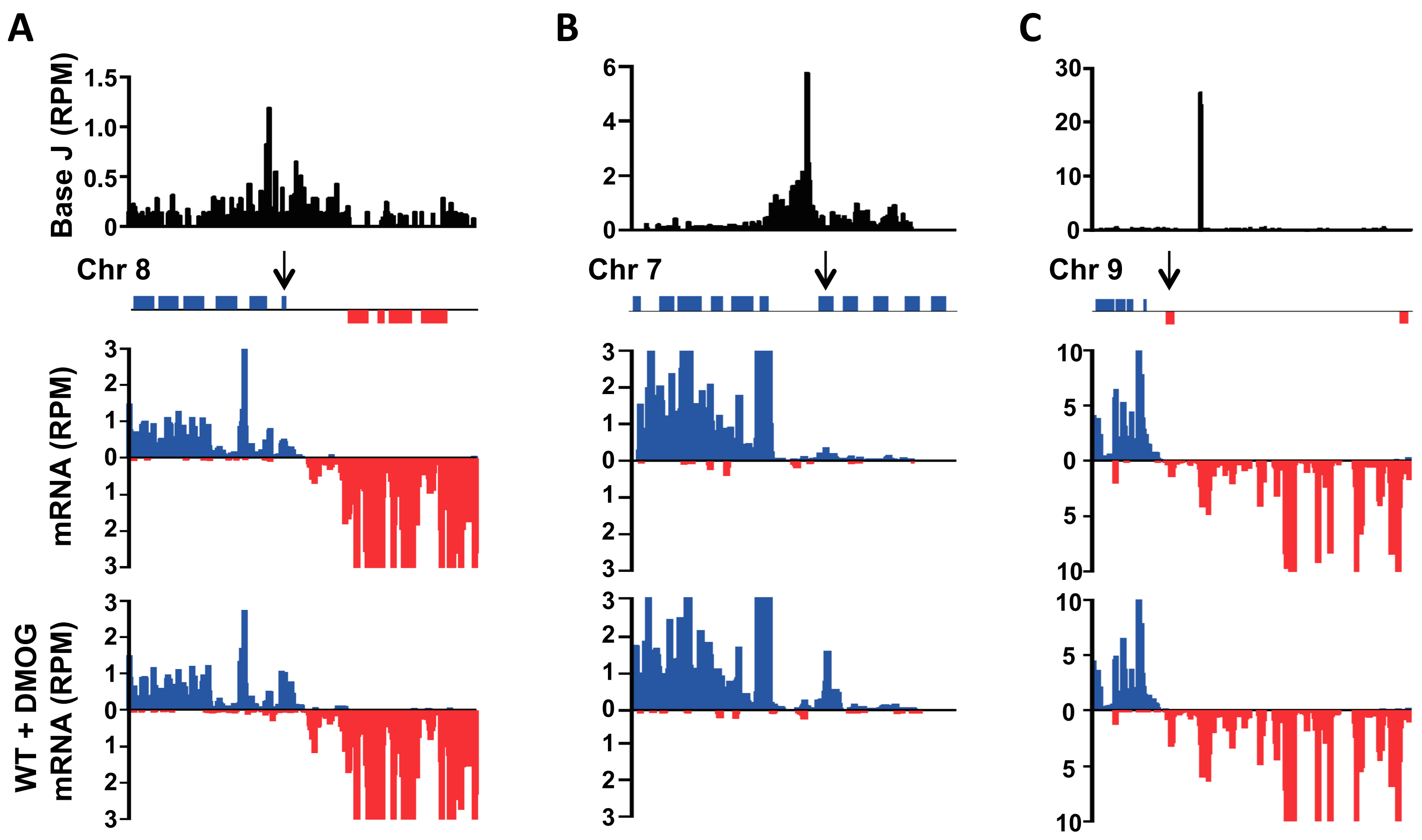

Supplemental Figure 7. Loss of base J results in up-regulated gene expression of downstream genes in *T. brucei*.
(A) A region on chromosome 8 from 540kb-570kb. Mapped reads from base J IP-seq are shown at the top, plotted as reads per million reads mapped (rpm). ORFs are illustrated below. Blue, genes on the top strand; red, genes on the bottom strand. Arrow indicates the gene found up-regulated in DMOG treated WT *T. brucei* by total RNA-seq (Tb427.08.1660). Total RNA-seq reads from DMSO treated WT and DMOG treated WT are plotted as rpm. Reads mapped to the top strand are shown in blue and reads mapped to the bottom strand in red.
(B) Same as in (A), but chromosome 7 from 1750kb-1780kb is shown. Arrow indicates gene Tb427.07.6500.
(C) Same as in (A), but chromosome 9 from 1120kb-1180kb is shown. Arrow indicates gene Tb427tmp.160.5350.

Supplemental Figure 8

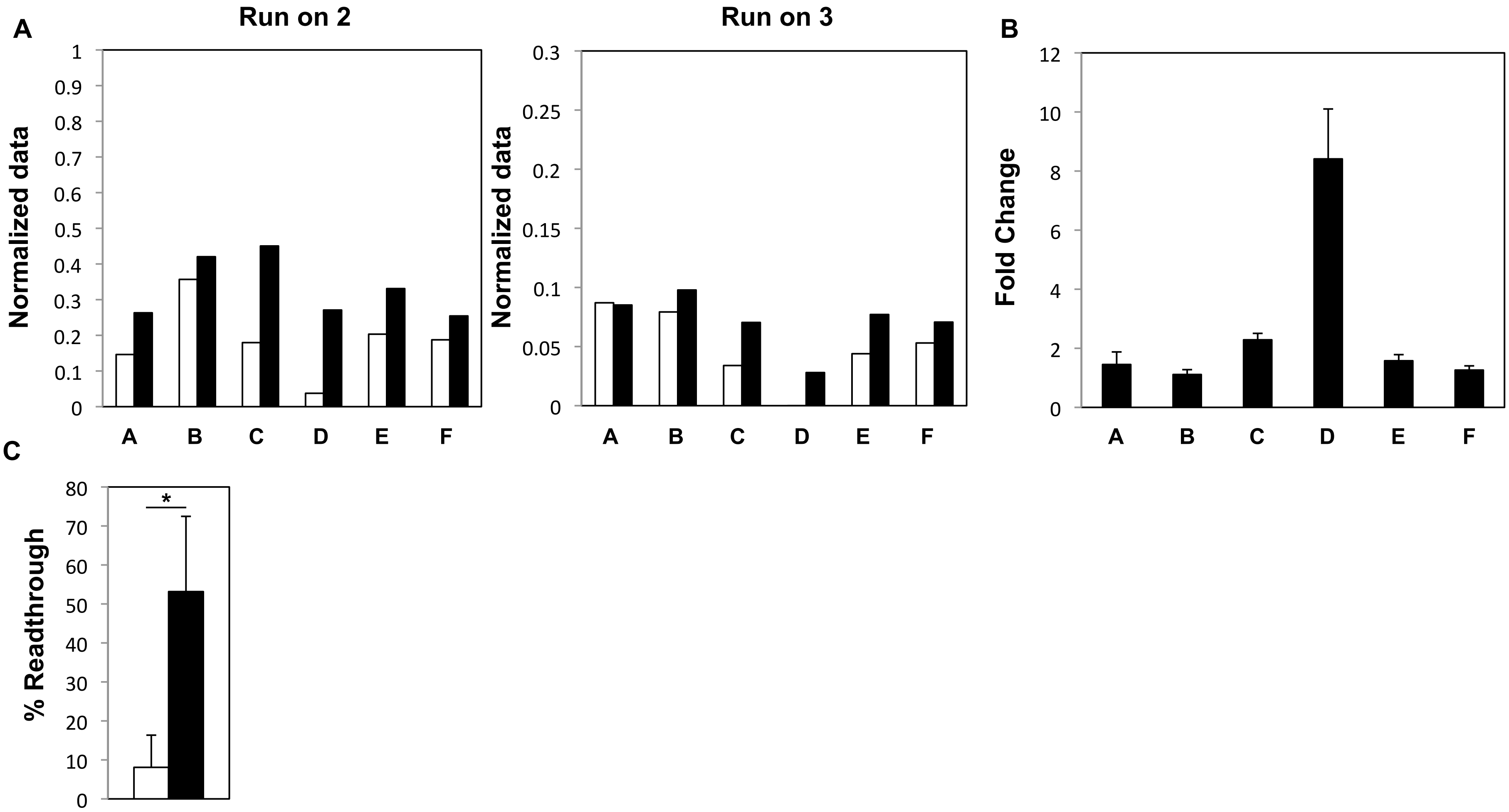

Supplemental Figure 8. Nuclear run on analysis.

(A) Normalized data from two additional independent run on experiments. White bars, WT *T. brucei*; Black bars, DMOG treated WT *T. brucei*. The probes used were the same as those shown in Figure 6A. Probe signal was normalized to SL, which was set to 1.

(B) The average fold change (DMOG treated WT normalized signal divided by WT *T. brucei* normalized signal) for three independent run on experiments. Error bars represent the fold change standard deviation of the three run on experiments with the exception of probe D. Fold change could not be determined for run on 3 probe D, shown in (A), given that the WT normalized signal was zero.

(C) The percent readthrough transcription for each experiment was determined by dividing the normalized signal for probe D by the upstream average normalized signal of probes A-C. White bar, WT *T. brucei*; Black bar, DMOG treated WT *T. brucei*. Error bars represent the standard deviation of three independent run on experiments. Asterisk indicates p-value < 0.05 by one-tailed Student's t-Test.

Supplemental Figure 9

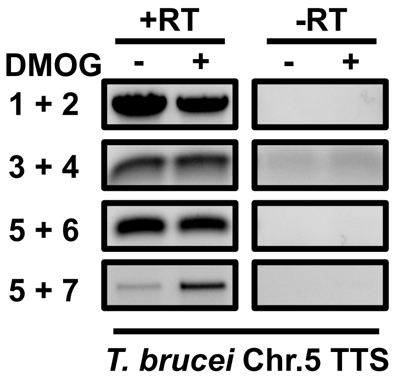

Supplemental Figure 9. Readthrough transcription in *T. brucei*.
WT and WT DMOG treated *T. brucei* cells were analyzed by single-strand RT PCR. The region upstream of gene Tb427.05.3990, which was found increased upon J loss, was analyzed. Primers correspond to the schematic shown in Figure 3A. Plus RT and minus RT controls are shown. -, DMSO; +, 1mM DMOG.

Supplemental Table 1

TTS	Chromosome	Left	Right	RNAs
3.3	3	257,889	260,160	T
4.1	4	125,231	134,259	
5.2	5	358,712	368,098	T
5.3	5	413,600	416,349	
6.3	6	496,623	499,407	
7.2	7	56,646	61,002	
8.1	8	391,471	395,193	
9.1	9	271,624	278,097	T&B
9.2	9	410,541	420,123	T&B
10.2	10	266,993	271,075	
12.1	12	175,105	177,453	
13.2	13	234,147	235,431	
14.1	14	157,511	166,986	
15.2	15	322,199	330,364	T&B
16.2	16	455,043	455,990	B
20.4	20	655,704	657,200	
21.2	21	165,335	167,157	B
21.3	21	448,056	449,719	T
22.2	22	10,320	11,184	
22.3	22	508,165	509,101	
23.2	23	224,857	228,092	T&B
24.3	24	619,452	623,933	T&B
25.2	25	421,375	423,905	
27.2	27	371,632	385,084	
27.3	27	715,532	718,771	
28.1	28	111,902	112,522	
28.2	28	587,431	595,609	
28.3	28	1,038,359	1,039,013	
29.2	29	651,387	656,655	
30.3	30	784,998	785,934	B
32.2	32	536,618	540,807	
33.5	33	806,005	806,444	
34.2	34	298,242	304,334	
34.3	34	469,757	471,821	T&B
35.4	35	640,557	646,831	
35.6	35	1,069,989	1,070,801	
36.2	36	489,603	495,890	T&B
36.3	36	1,032,191	1,035,959	T&B
36.7	36	1,885,279	1,886,719	

Supplemental Table 2

Library #	1	2	3	4	5	6
Species	T. brucei	T. brucei	T. brucei	T. brucei	L. major	L. major
treatment	DMSO	DMOG	DMSO	DMOG	DMSO	DMOG
Type of RNA sequenced	small RNA	small RNA	polyA enriched RNA (mRNA)	polyA enriched RNA (mRNA)	small RNA	small RNA
Genome used for alignment	T. brucei 427 v6.0	T. brucei 427 v6.0	T. brucei 427 v6.0	T. brucei 427 v6.0	L. major v4.2	L. major v4.2
Minimum read length considered for alignment (nt)	18	18	n/a	n/a	18	18
Total reads (millions)	43.0	37.6	30.2	26.9	21.2	65.2
Overall (unique and non unique) alignment rate %	97.12	97.49	95.87	95.40	97.72	97.99
